# Supplementary material for: Stacking Model‐Based Classifiers for Dealing With Multiple Sets of Noisy Labels
Source: Biom J. 2025 Mar 12;67(2):e70042. doi: 10.1002/bimj.70042 (PMC11898607; doi:10.1002/bimj.70042)
Supplement: Supplementary file 1 — Supporting Information [file BIMJ-67-e70042-s001.zip › density-based-ensemble-model-main/competitors_real_data/netlab-master/netlab-master/help/conffig.htm]

Netlab Reference Manual conffig


# conffig

## Purpose

Display a confusion matrix.

## Synopsis

```
conffig(y, t)
fh = conffig(y, t)
```

## Description

`conffig(y, t)` displays the confusion matrix
and classification performance for the predictions mat{y}
compared with the targets `t`. The data is assumed to be in a
1-of-N encoding, unless there is just one column, when it is assumed to
be a 2 class problem with a 0-1 encoding. Each row of `y` and `t`
corresponds to a single example.

In the confusion matrix, the rows represent the true classes and the
columns the predicted classes.

`fh = conffig(y, t)` also returns the figure handle `fh` which
can be used, for instance, to delete the figure when it is no longer needed.

## See Also

`confmat`, `demtrain`

---

**Pages:**
Index


---

Copyright (c) Ian T Nabney (1996-9)
